# Successful Treatment of Median Arcuate Ligament Syndrome in the Hybrid Operating Room: A Report of Four Cases

**DOI:** 10.7759/cureus.87713

**Published:** 2025-07-11

**Authors:** Shinji Yamaguchi, Ryosuke Nakano, Naruhiko Honmyo, Hiroshi Sakai, Shintaro Kuroda, Hiroyuki Tahara, Masahiro Ohira, Kentaro Ide, Keigo Chosa, Kazuo Awai, Tsuyoshi Kobayashi, Hideki Ohdan

**Affiliations:** 1 Department of Gastroenterology and Transplant Surgery, Hiroshima University, Hiroshima, JPN; 2 Department of Diagnostic Radiology, Hiroshima University, Hiroshima, JPN

**Keywords:** abdominal pain, celiac artery, hybrid technique, median arcuate ligament syndrome, surgical repair

## Abstract

Median arcuate ligament syndrome (MALS) is a rare disease characterized by abdominal pain attributed to the compression of the celiac trunk by the median arcuate ligament (MAL). In this study, we describe four patients with MALS who were successfully treated using surgical dissection of the MAL in a hybrid operating room that allowed intraoperative angiography. All four patients described in this report experienced abdominal pain. In three of these patients, MALS manifested as a consequence of rupture of a superior pancreaticoduodenal artery (PDA) aneurysm. The remaining patient was diagnosed with an asymptomatic PDA aneurysm through examination. The three cases of ruptured PDA aneurysms in our study required emergency embolization with intraoperative vascular radiology prior to the surgical dissection of the MAL. The standard therapeutic approach for MALS involves decompression of the celiac trunk by dissection of the MAL to obtain sufficient antegrade blood flow from the celiac artery (CA) to the proper hepatic artery. It is difficult to evaluate the efficacy of decompression of the CA using surgery alone because hemodynamic changes cannot be monitored intraoperatively. The surgeries were performed in a hybrid operating room, which contained intraoperative angiography and computed tomography equipment. It facilitated the evaluation of blood flow from the celiac trunk in real time, allowing the intraoperative verification of augmented blood perfusion within the CA, subsequent to the transection of the MAL. The postoperative course was uneventful, and none of the patients experienced any symptoms related to MALS after surgery. We describe the treatment of four patients who underwent successful hybrid surgery for MALS. Intraoperative angiography provided reliable information to evaluate the efficacy of the surgical division of the MAL.

## Introduction

The median arcuate ligament (MAL) is an arch-like tendinous fascial band formed by the convergence of the right and left diaphragmatic crura, encircling the aortic hiatus, which is typically situated at the level of the twelfth thoracic vertebra. Under normal anatomical conditions, the MAL lies superior to the origin of the celiac artery (CA) as it branches off from the descending aorta [1,2]. Median arcuate ligament syndrome (MALS) is an uncommon disorder marked by abdominal pain caused by the MAL exerting pressure on the celiac trunk [1,3,4]. Varying degrees of celiac trunk compression reportedly occur in 10-50% of the general population [5,6]. However, the incidence of symptomatic MALS is 0.4%, and the majority of patients remain asymptomatic [4,7]. On physical examination, patients may exhibit slight abdominal tenderness, and in rare cases, a bruit can be heard over the abdomen. The clinical signs and symptoms support the diagnosis of MALS based on exclusion and the demonstration of CA narrowing or obstruction using diverse imaging techniques [2].

The standard therapeutic approach for MALS involves decompression of the CA by dissection of the MAL, which is increasingly being accomplished laparoscopically because of its minimally invasive approach. However, because of the deep anatomical location of the retroperitoneum, laparoscopic identification of the CA's root is difficult and leads to a high rate of conversion to open surgery [2,4,8,9].

Hybrid operating rooms are equipped with advanced angiographic imaging systems for complex and delicate interventions. The featured operating room was equipped with angiography equipment (Artis zee biplane system®, Siemens Healthineers, Erlangen, Germany) and a computed tomography (CT) scanner (SOMATOM Definition AS Open - RT Pro edition®, Siemens Healthineers). The Artis zee biplane system exhibits excellent performance in terms of imaging and position flexibility. Moreover, the SOMATOM Definition AS Open - RT Pro edition serves as a valuable tool for providing real-time imaging to assess the outcomes of interventions during surgical procedures [10]. Herein, we describe four patients with MALS who were successfully treated in a hybrid operating room.

## Case presentation

Case 1

A 57-year-old woman was taken to her local hospital by ambulance because of sudden upper abdominal pain. Contrast CT revealed retroperitoneal hemorrhage, and angiography showed stenosis of the CA and a rupture of the anterior superior pancreaticoduodenal artery aneurysm (PDAA). The patient was diagnosed with MALS and ruptured PDAA and was referred to our hospital for treatment of the hemorrhage. Coil embolization of the aneurysm was immediately performed. The patient was diagnosed with MALS and underwent elective MAL division.

Case 2

A 51-year-old woman was admitted to her local hospital with sudden lower abdominal pain. Contrast CT revealed abdominal hemorrhage, while angiography identified stenosis of the CA and an aneurysm in the collateral circulation between the middle colic artery and the dorsal pancreatic artery, which was the source of the bleeding. She was diagnosed with MALS and a ruptured aneurysm and referred to our hospital for treatment. After coil embolization of the abdominal aneurysm, the patient underwent elective surgery for MALS.

Case 3

A 56-year-old man presented with sudden-onset upper abdominal pain. Contrast CT and angiography revealed stenosis of the CA and a 9 mm inferior PDAA. He was diagnosed with MALS and referred to our hospital for surgery. He underwent elective surgery for MALS. We did not perform preoperative endovascular treatment for the aneurysm because it was small and expected to reduce in size after the MAL division.

Case 4

A 47-year-old man was admitted to a local hospital with hemorrhagic shock. Enhanced CT showed massive abdominal hemorrhage. Angiography revealed a ruptured anterior PDAA and stenosis of the CA. Coil embolization of the ruptured PDAA was performed, and the patient was transferred to our hospital for intensive care. After arrival at our hospital, angiography revealed active bleeding from the PDAA, and coil embolization of the inferior PDA was performed. On the same day, after endovascular treatment, the patient began to develop abdominal compartment syndrome due to increased intra-abdominal pressure, and surgery was performed to remove a large hematoma. Owing to intestinal edema, we could not close the abdomen and performed temporary abdominal closure using a negative pressure therapy system (3MTM AbTheraTM Advance, Solventum, St Paul, MN, USA). The abdomen was closed 10 days after the secondary surgery. The patient was diagnosed with MALS, and elective surgery for division of the MAL was planned.

**Figure 1:**
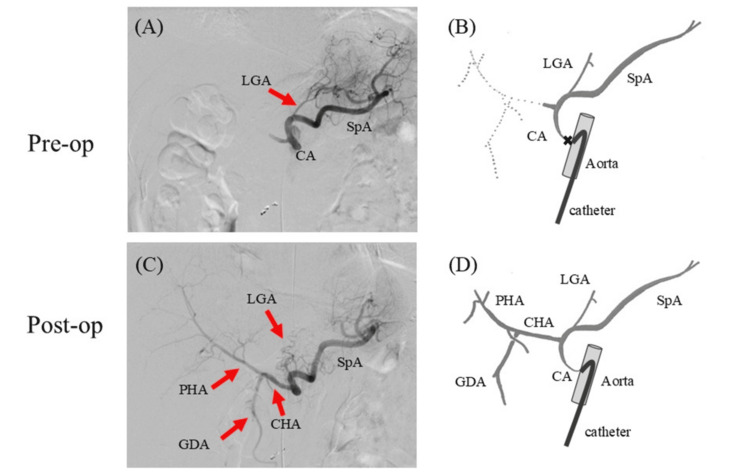
Case 1 In the pre-median arcuate ligament (MAL) division angiography of Case 1, (A, B) blood flow to the proper hepatic artery (PHA) from the celiac artery (CA) is not observed due to collateral flow from the superior mesenteric artery (SMA) caused by severe celiac trunk stenosis. (C, D) After MAL release, antegrade blood flow to the PHA and gastroduodenal artery (GDA) from the CA is restored. Pre-op, pre-operation; Post-op, post-operation; CHA, common hepatic artery; LGA, left gastric artery; SpA, splenic artery

**Figure 2:**
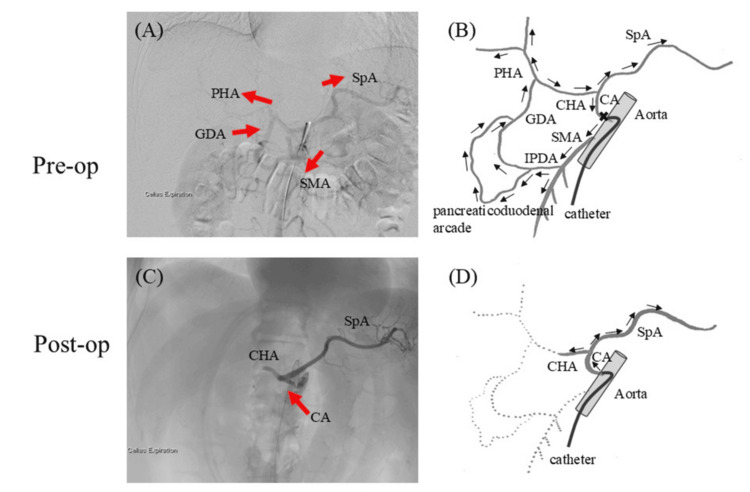
Case 3 In pre-median arcuate ligament (MAL) division angiography of Case 3, (A, B) blood flow from the celiac artery (CA) is not observed due to severe celiac trunk stenosis. However, retrograde flow to the splenic artery (SpA) and the proper hepatic artery (PHA) from the superior mesenteric artery (SMA) via the pancreaticoduodenal arcade is observed. (C, D) After MAL release, antegrade blood flow to the common hepatic artery (CHA) and SpA is restored. Pre-op, pre-operation; Post-op, post-operation; GDA, gastroduodenal artery; IPDA, inferior pancreaticoduodenal artery

In the above four patients, we performed open surgery for the MAL division in a hybrid operating room to check the blood flow of the CA intraoperatively. We performed angiography of the intraabdominal arteries before and after the division of the MAL and confirmed, in real time, that the division of the MAL had resulted in improved celiac stenosis and blood flow in the CA. In Case 1, blood flow to the proper hepatic artery from the CA was not observed because of the flow from the superior mesenteric artery (SMA) due to severe celiac trunk stenosis in the angiography of the pre-MAL division. When the MAL had been released, antegrade blood flow to the proper hepatic artery (PHA) was observed (Figure 1). In Case 3, blood flow in the CA was not observed due to severe celiac trunk stenosis, and retrograde flow of the splenic artery and PHA was observed from the SMA through the pancreaticoduodenal arcade in pre-MAL division angiography. When the MAL had been released, antegrade blood flow to the CHA and splenic artery was observed (Figure 2). In Case 2, the pressure gradient of the CA was measured during inspiration and expiration. Prior to the MAL division, it measured 5 mmHg; however, following the release, it improved to 2 mmHg. No intraoperative complications were observed in any of the four patients. The postoperative length of hospital stay was 5-10 days, and only one case of mild liver dysfunction (Clavien-Dindo Classification Grade I [11]) was observed as a postoperative complication (Table 1). A follow-up CT performed one week after surgery revealed an improvement in the rate of CA stenosis compared with that observed preoperatively in all patients. In Case 3, follow-up CT revealed a reduction in the size of the PDAA (from 9 to 6 mm) (Table 2). Although the follow-up period varied depending on the case, none of the patients presented with symptoms related to MALS during the six to nine-month follow-up period.

**Table 1:** Summary of the patient characteristics in the four cases *The classification of complications is based on the Clavien–Dindo Classification [11]

Case	Operative Time (min)	Blood Loss (mL)	Intraoperative Complications	Postoperative Hospital Stay (days)	Postoperative Complications
1	205	148	-	10	Liver dysfunction (Grade 1)*
2	140	39	-	9	-
3	145	33	-	5	-
4	178	76	-	7	-

**Table 2:** Summary of the surgical outcomes of the four cases Each PDAA diameter was measured by contrast-enhanced computed tomography scan six months after surgery. Although there were differences in the follow-up periods, the four patients underwent follow-up examinations for six to nine months, and none of them experienced a recurrence of symptoms. CA, celiac artery; PDAA, pancreaticoduodenal artery aneurysm; Pre-op, pre-operation; Post-op, post-operation

Case	CA Stenosis Rate (%)(minimum diameter / maximum diameter mm)	PDAA Diameter (mm)	Post-operative Symptoms
	Pre-op → Post-op	Pre-op → Post-op	
1	80(1/5) → 20(4/5)	50 → -	-
2	57(3/7) → 23(5/7)	7 → -	-
3	67(2/6)→ 33(4/6)	9 → 6	-
4	43(4/7) → 28(5/7)	26 → -	-

## Discussion

Here, we describe four cases of successful surgical treatment of CA compression syndrome in a hybrid operating room. Evaluation of the efficacy of CA decompression is generally difficult using surgery alone because hemodynamic changes cannot be monitored intraoperatively. The hybrid operating room used in this study contained intraoperative angiography and CT devices [10]. This equipment facilitated the evaluation of blood flow from the celiac trunk in real time and intraoperative verification of augmented blood perfusion within the CA after the division of the arcuate ligament [4].

The clinical manifestations of MALS are highly variable. It most commonly affects middle-aged women, who typically exhibit a combination of postprandial upper abdominal pain, unintended weight loss, and the presence of an abdominal bruit on examination. These symptoms are frequently attributed to vascular insufficiency, which can lead to intestinal angina [2].

The pain often worsens with physical exertion and may be associated with gastrointestinal symptoms such as abdominal bloating, nausea, vomiting, or diarrhea [2,12]. Abdominal pain tends to worsen during exhalation and may be relieved when the patient adopts a knee-chest posture. This increase in pain during expiration is thought to result from the diaphragm's upward shift, which stretches the diaphragmatic crura and increases tension on the MAL. Consequently, individuals with anatomical susceptibility may experience vascular compression during this phase of respiration [2]. The four patients we reported here were all diagnosed with MALS, triggered by abdominal pain.

All four patients experienced abdominal pain. In three of these patients, MALS manifested as a consequence of the rupture of a PDAA. The remaining patient was diagnosed with an asymptomatic PDAA through the examination. Aneurysms are uncommon, constituting only 2% of all visceral aneurysms [13]. However, PDAAs are among the most life-threatening of all splanchnic aneurysms, with a mortality rate of up to 50% in the event of an aneurysmal rupture. Accumulating evidence indicates that 50-80% of PDAAs are associated with CA stenosis [14-17]. Hemodynamic mechanisms of aneurysm formation in the collateral vessels between the CA and SMA have been reported. When the CA is compressed, blood flow to the liver, spleen, and stomach is maintained through collateral circulation from the SMA via the PDA. Prolonged increased flow and hemodynamic stress within this collateral network are thought to weaken the arterial wall over time, promoting vessel dilation, tortuosity, and eventually aneurysm formation [4,18-21]. Once a PDAA is diagnosed, it should be treated promptly, because a ruptured aneurysm is life-threatening [4,22]. The three cases of ruptured PDAAs in our study required emergency embolization with intraoperative vascular radiology prior to the surgery involving division of the MAL.

Ensuring adequate forward flow from the CA to the PHA is a critical goal of this surgical intervention [4]. Treatment approaches have evolved to include minimally invasive options. Nevertheless, owing to the retroperitoneum's deep anatomical location, laparoscopic identification of the CA's root remains challenging, resulting in a conversion rate to open surgery of approximately 10% [8,9,23]. Therefore, prioritizing safety, an open surgical approach was chosen. Both open and laparoscopic approaches demonstrate symptom relief in over 70% of adult and pediatric patients. However, some patients require additional interventions or experience symptom recurrence [8,24,25]. Some studies recommend percutaneous transluminal angioplasty for patients experiencing recurrent or persistent symptoms [26-28]. While endovascular stenting alone has shown promise in severe cases, it is generally not recommended as the sole treatment due to high failure rates [29,30]. In all our cases, hybrid surgery resulted in complete resolution of symptoms postoperatively, with no recurrence observed during follow-up.

Although our patients could have undergone surgery using the C-arm in a common operating room, hybrid surgery provides further information regarding the improvement of direct blood flow to the CA intraoperatively. First, digital subtraction angiography provides high-quality imaging of the intraabdominal vessels. Second, the radiologists can perform a targeted contrast-enhanced evaluation of the celiac axis and SMA to confirm whether MAL release would be effective. Third, in the event of bleeding resulting from a CA injury, bleeding can be controlled through the application of balloon occlusion. Consequently, hybrid surgery makes MAL release safe and effective. In all four patients, the surgery was performed successfully without any intraoperative complications. Postoperative CT revealed improvements in the areas of stenosis in each case. Furthermore, there have been no reports of the postoperative recurrence of abdominal symptoms.

## Conclusions

Herein, we report four cases of MALS in which the patients were successfully treated in a hybrid operating room. The standard therapeutic approach for MALS involves decompression of the CA by division of the MAL to obtain sufficient antegrade blood flow from the CA to the proper hepatic artery. The hybrid operating room facilitates a real-time assessment of blood flow in the CA during surgery, akin to a highly efficient navigator. Consequently, this approach offers a secure and reliable surgical intervention for MALS.
